# Deficits in proprioception and strength may contribute to the impaired postural stability among individuals with functional ankle instability

**DOI:** 10.3389/fphys.2024.1342636

**Published:** 2024-03-01

**Authors:** Yanhao Liu, Shiyu Dong, Qi Wang, Ziyin Liu, Qipeng Song, Peixin Shen

**Affiliations:** ^1^ College of Sports and Health, Shandong Sport University, Jinan, China; ^2^ College of Sports Human Sciences, Beijing Sport University, Beijing, China

**Keywords:** ankle sprain, functional ankle instability, postural control, kinesthesia, jump landing

## Abstract

**Purpose:** The correlations of postural stability with proprioception and strength may explain the recurrent sprains among individuals with functional ankle instability (FAI). This study aimed to compare anterior-posterior (AP) and medial-lateral (ML) postural stability, along with ankle proprioception and strength between individuals with and without FAI and investigated their correlations.

**Methods:** Forty participants with FAI and another 40 without FAI were recruited. Their postural stability, represented by time to stabilization (TTS) in the AP (TTS_AP_) and ML (TTS_ML_) directions, was calculated by the ground reaction force during jumping onto a force plate. Their ankle proprioception and strength during plantarflexion/dorsiflexion and inversion/eversion were measured using a proprioception device and a strength testing system, separately.

**Results:** Individuals with FAI had longer TTS_AP_ (*p* = 0.015) and TTS_ML_ (*p* = 0.006), larger ankle proprioception thresholds (*p* = 0.000–0.001), and less strength (*p* = 0.001–0.017) than those without FAI. Correlations between strength and TTS_AP_ were detected among individuals with (ankle plantarflexion, r = −0.409, *p* = 0.009) and without FAI (ankle plantarflexion, r = −0.348, *p* = 0.028; ankle dorsiflexion, r = −0.473, *p* = 0.002). Correlations of proprioception (ankle inversion, r = 0.327, *p* = 0.040; ankle eversion, r = 0.354, *p* = 0.025) and strength (ankle eversion, r = −0.479, *p* = 0.002) with TTS_ML_ were detected among individuals without FAI but not among those with FAI.

**Conclusion:** Individuals with FAI have worse postural stability and proprioception and less strength. Their proprioception and strength decreased to a point where they could not provide sufficient functional assistance to the ML postural stability. Improvements in proprioception and strength may be keys to prevent recurrent ankle sprains among individuals with FAI.

## 1 Introduction

Ankle sprain has the highest recurrence rate among all lower-extremity musculoskeletal injuries (Fong et al., 2007; Kerr et al., 2022), with an annual economic burden of approximately $6.2 billion in the United States (Gribble et al., 2016). Following an initial sprain, 40% of individuals develop functional ankle instability (FAI), characterized by recurrent sprains, sensorimotor impairments, experiences of “giving way,” and feelings of instability at the ankle (Arnold et al., 2009; Herzog et al., 2019; Huang et al., 2021).

Postural stability deficit is one of the strongest risk factors for ankle sprain (Hertel and Corbett, 2019). Owing to the biomechanical contribution of the ankle joint on jump performance (Giustino et al., 2022), jump landing test is one of the most common functional tests to assess postural stability in the anterior-posterior (AP) and medial-lateral (ML) directions among individuals with FAI (Ross et al., 2009). Postural stability during jump landing is usually measured in terms of time to stabilization (TTS) in the AP (TTS_AP_) and ML (TTS_ML_) directions, which represents the time needed to stabilize the body (McCann et al., 2018).

Maintaining postural stability involves the integration of sensory input with information from the musculoskeletal systems (Grassi et al., 2018). Proprioception and strength are two crucial contributors in maintaining postural stability and preventing sprains during sports and daily activities. Proprioceptive receptors transmit information about the position of limbs and body segments, as well as the velocity and direction of their movements, to the central nervous system to guarantee smooth and coordinated body movements during balance control (Xue et al., 2021). Sufficient torque generated by lower limb muscles ensures rapid adjustments when countered with unexpected disturbances (Park et al., 2019; Khalaj et al., 2021).

Previous studies investigated the correlations of postural stability with proprioception (Santos and Liu, 2008) and muscle strength (McCann et al., 2018). However, they did not differentiate postural stability in the AP and ML directions, and most of them focused only on individuals with FAI but not those without FAI. Humans adopt different mechanisms to maintain AP and ML postural stability (O'Connor and Kuo, 2009). Compared with AP postural stability, ML postural stability is harder to maintain and depends on motor control through active foot placement and integrated sensory feedback, such as proprioception (O'Connor and Kuo, 2009; Rankin et al., 2014). The majority of ankle sprains occur in the ML direction (primarily during ankle inversion) (Herzog et al., 2019), making it particularly important to differentiate between AP and ML postural stability. To the best of our knowledge, no studies have investigated the correlations of proprioception and strength with the AP and ML postural stability among individuals with and without FAI. Distinguishing AP and ML postural stability and investigating their correlations with proprioception and strength among individuals with and without FAI can help deepen our understanding of the causes of postural stability deficits and recurrent ankle sprains and provide guidance for the rehabilitation and prevention of ankle sprains.

This study aimed to compare AP and ML postural stability, proprioception, and strength between individuals with and without FAI and investigate the correlations of proprioception and strength with AP and ML postural stability. Addressing these issues can help select targeted interventions to enhance postural stability and develop precise ankle sprain prevention approaches among individuals with FAI. Our hypothesis are as follows: 1. Individuals with FAI have worse AP and ML postural stability, impaired proprioception, and less strength than those without FAI; 2. AP and ML postural stability are significantly correlated with proprioception and strength among individuals with and without FAI.

## 2 Material and methods

### 2.1 Design

This study has a cross-sectional design. Before participation, all participants read and signed informed consent approved by the Institutional Review Boards of Shandong Sport University (2022001).

### 2.2 Participants

The sample size was estimated using G*Power software (Version 3.1) based on two studies: one detected an effect size = 0.80 in TTS between people with and without FAI (with = 1.86 ± 0.67s, without = 1.44 ± 0.33s) (Ross et al., 2009), and another detected an *r* ^2^ = 0.25 between muscle strength and postural stability (McCann et al., 2017). A minimum of 52 and 66 participants must be recruited to guarantee an α of 0.05 and statistical power of 0.80.

Participants were recruited from a local university from May to July 2023 through e-newsletters, e− and paper notifications, and e-mail. After the purpose and process of the study were introduced to the participants, 124 people were willing to enroll in the study, of whom 58 had self-reported FAI symptoms and 66 had no FAI symptoms. The eligible participants were screened according to the inclusion and exclusion criteria of the study. Inclusion criteria for participants with FAI were as follows: (1) at least one severe ankle sprain resulting in pain, swelling, and activity limitation for at least 1 day within at least 12 months before the start of this study; (2) age 18–25 years; (3) more than two times of ankle “giving way,” which refers to the feeling of uncontrollable or unpredictable excessive inversion of the ankle (Delahunt et al., 2010; Gribble et al., 2014), in the past 6 months; (4) persistent ankle instability and dysfunction during daily activities, which refers to the feeling of ankle instability during daily living and sporting activities (Delahunt et al., 2010) or the experience of difficulty in certain daily activities such as putting on shoes, getting in and out of a car, due to pain or joint instability (Lentell et al., 1995); (5) with a score ≤24 on the Cumberland Ankle Instability Tool (CAIT) (Gribble et al., 2014). Inclusion criteria for participants without FAI were as follows: (1) no previous ankle sprain/injury and no giving way or instability and (2) CAIT score ≥28. Exclusion criteria for all participants were as follows: (1) mechanical instability indicated by positive findings of talar tilt and anterior drawer test; (2) a fracture or surgical procedure to the lower extremity; (3) acute injury of the lower extremity within the last 3 months; (4) neurological disease, diabetes, and vestibular disorders; (5) bilateral FAI.

Following eligibility assessment, 40 individuals with FAI and another 40 without FAI were recruited. Their age, height, body mass, CAIT score, and ankle sprain and giving way experience are shown in Table 1. The number of left-dominant and right-dominant individuals (2 left and 38 right) was equal in each group. The dominant limb was defined as the limb each participant used during kicking a ball (Mitrousis et al., 2023).

**TABLE 1:** Participants’ baselines.

Group	FAI	Non-FAI	*p*-value
	(n = 40, female = 13)	(n = 40, female = 13)	
Age (years)	21.3 ± 1.3	22.4 ± 2.2	.098
Height (cm)	173.7 ± 8.7	173.9 ± 8.2	.931
Body mass (kg)	66.1 ± 10.2	66.7 ± 9.6	.809
CAIT (score)	16.9 ± 3.5	29.3 ± 0.6	<.001
No. previous ankle sprains (times)	2.9 ± 1.1	-	-
No. giving way episodes (times)	6.1 ± 1.6	-	-

CAIT: cumberland ankle instability tool.

### 2.3 Protocol

The CAIT scores, history of ankle sprain, and experience of giving way were recorded before the tests. Postural stability and proprioception were measured in a random order. Strength was assessed lastly to avoid fatigue. The individuals with FAI were tested on their affected limbs, including 26 dominant and 14 nondominant limbs. Meanwhile, 26 dominant and 14 nondominant limbs were tested among the individuals without FAI.

All participants’ CAIT scores were assessed using a paper-based questionnaire by a qualified physiotherapist with 6 years of clinical experience. The remaining three tests were conducted by three testers. For consistency, each test was conducted by the same tester. All data were collected in the Lab of Biomechanics at Shandong Sport University from May to July 2023.

### 2.4 Postural stability test

**FIGURE 1:**
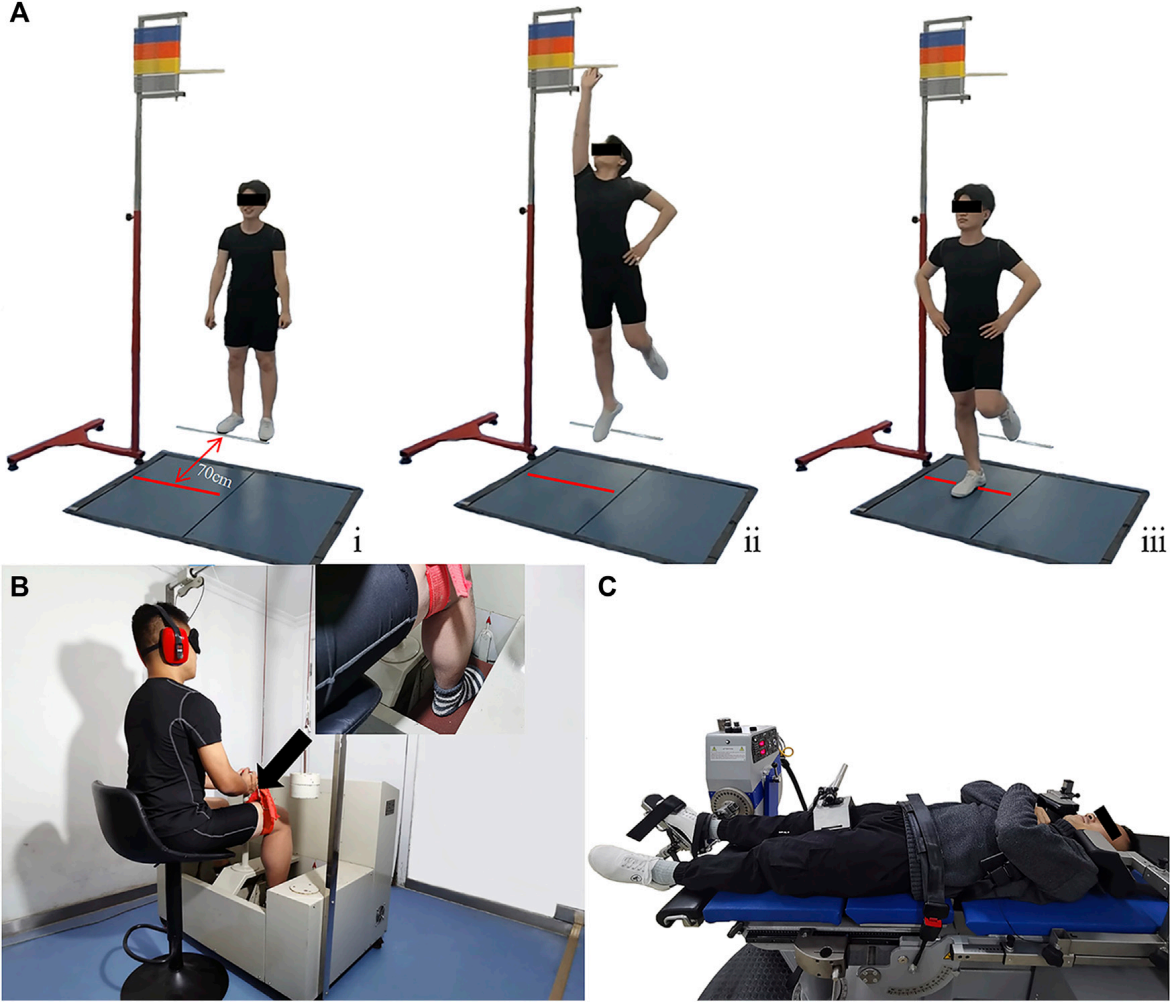
Test illustrations. **(A)** Jump landing test. **(B)** Proprioception test. **(C)** Strength test.

Postural stability was assessed by a jump landing test. The participants stood 70 cm away from the center of a force plate (AMTI, AMTI Inc., Watertown, MA, US) (Figure 1A ⅰ) and jumped forward and upward with both legs and landed onto the force plate (Gribble et al., 2010). During the jump, the participants touched the target on the vertical jump tester to ensure a jump height (Figure 1A ⅱ) and landed on their tested limbs. After landing, the participants maintained stability for 5 s (Figure 1A ⅲ). This test demonstrated good reliability (Fransz et al., 2016). Three trials were recorded, with a minimum interval of 60 s in between.

Jump height was determined by a vertical jump test before the jump landing test. Each participant stood with both feet next to a vertical jump tester (Guangzhou Gaia Sports Goods Co., ltd, China) and stretched his/her upper limbs upward as high as possible. Their standing stretching height (H_1_) was measured. He/she jumped as high as possible to touch a high point (H_2_). The jump height was calculated using the following formula:

$$\text{Jump height}=\left(H_{2}‐H_{1}\right)/2+H_{1\;}$$ (1)

### 2.5 Proprioception test

Proprioception was tested using a proprioception device (Sunny, Jinan, China) (Figure 1B) and indicated by the ankle passive detection sense, which has shown good test–retest reliability (ICC = 0.74–0.94) (Sun et al., 2015). This device comprises an operating platform and two pedals. The participants sat in a height-adjustable chair with both feet positioned on the testing pedal, hips and knees flexed at a 90° angle, and ankles in a neutral position. The participants wore eye masks and noise-canceling headphones throughout the tests to minimize distractions. During each test, one of the pedals rotated at an angular velocity of 0.4°/s, inducing ankle plantarflexion/dorsiflexion or inversion/eversion. As soon as the passive motion was perceived, the participants immediately pressed a hand-held switch to stop the pedal (Song et al., 2021). The proprioception threshold was defined as the angle of pedal rotation when the passive motion was perceived. Three trials were recorded in each direction, with a minimum interval of 60 s in between.

### 2.6 Strength test

Isokinetic strength during ankle plantarflexion/dorsiflexion and inversion/eversion was measured at an angular velocity of 60°/s by a strength testing system (IsoMed 2000; D. and R. Ferstl GmbH, Hemau, Germany) (Figure 1C), which showed good test–retest reliability (Gonosova et al., 2018). During the plantarflexion/dorsiflexion tests, the participants lay supine with their thighs, buttocks, and torsos fixed to the chair, lateral malleolus of the tested limbs aligned with the axis of rotation of the dynamometer arm, and started at 15° of dorsiflexion and ended at 35° of plantarflexion. During the inversion/eversion tests, the participants lay semiprone with the seat reclining at 45°, hips, knees, and ankles of the tested limbs flexed at 80°, 110°, and 10°, and started at 25° of ankle eversion and ended at 20° of ankle inversion. All the tests were conducted with consistent verbal commands and encouragement provided to all the participants. Three trials were recorded in each direction, with a minimum interval of 120 s in between.

### 2.7 Data processing

In the postural stability test, the ground reaction force (GRF) in AP and ML directions was recorded at 1000 Hz and then filtered using a fourth-order low-pass Butterworth filter with a cut-off frequency of 12 Hz (McCann et al., 2018). The filtered data, obtained from the time of landing (>10 N) to 5 s post-landing, were used to calculate TTS calculation through sequential average as follows (Fransz et al., 2015):

$$\text{Sequential Average }x\left(n\right)=\sum_{n=1}^{1000}\text{Fx}/n$$ (2)

$$\text{Sequential Average }y\left(n\right)=\sum_{n=1}^{1000}\text{Fy}/n$$ (3)

where Fx and Fy are the GRF in the AP and ML directions. TTS was defined as the time from landing to the sequential average of each component reached and remained within one-fourth of the standard deviation of the overall average (Fransz et al., 2016). In the proprioception and strength tests, the mean values in each direction of three trials were used for statistical analysis. In the strength test, the joint torques were normalized by body mass.

### 2.8 Statistics

Shapiro-Wilk tests were used to assess the normality of data distribution. Descriptive analysis was applied to examine outcome variables’ mean and standard deviations. Independent t-tests (normal distribution) or Mann–Whitney U tests (nonnormal distribution) were utilized to compare differences between individuals with and without FAI. Effect sizes (Cohen’s *d* for normal distribution, ɳ^2^ for nonnormal distribution) were used to evaluate the magnitude of between-group differences. Pearson (normal distribution) or Spearman correlations (nonnormal distribution) were adopted to determine the correlations of proprioception and strength to TTS while controlling for covariates (gender, age, height, and weight). Separate exploratory factor analysis was then carried out among each category of the variables of interest. Multivariable linear regression was used to explore the degree of correlation between each generated factor and TTS while controlling for the above-mentioned covariates. The thresholds for Cohen’s *d* were as follows: <0.20, trivial; 0.21–0.50, small; 0.51–0.80, medium; and >0.81, large. The thresholds for ɳ^2^ were as follows: 0.01–0.059, small; 0.06–0.14, medium; and >0.14, large. The thresholds for correlation coefficient were as follows: 0–0.1 (trivial), >0.1–0.3 (weak), >0.3–0.5 (moderate), and >0.5 (strong) (Cohen, 1988). All analyses were conducted in SPSS Version 26.0 (IBM; Armonk, NY, United States).

## 3 Results

**FIGURE 2:**
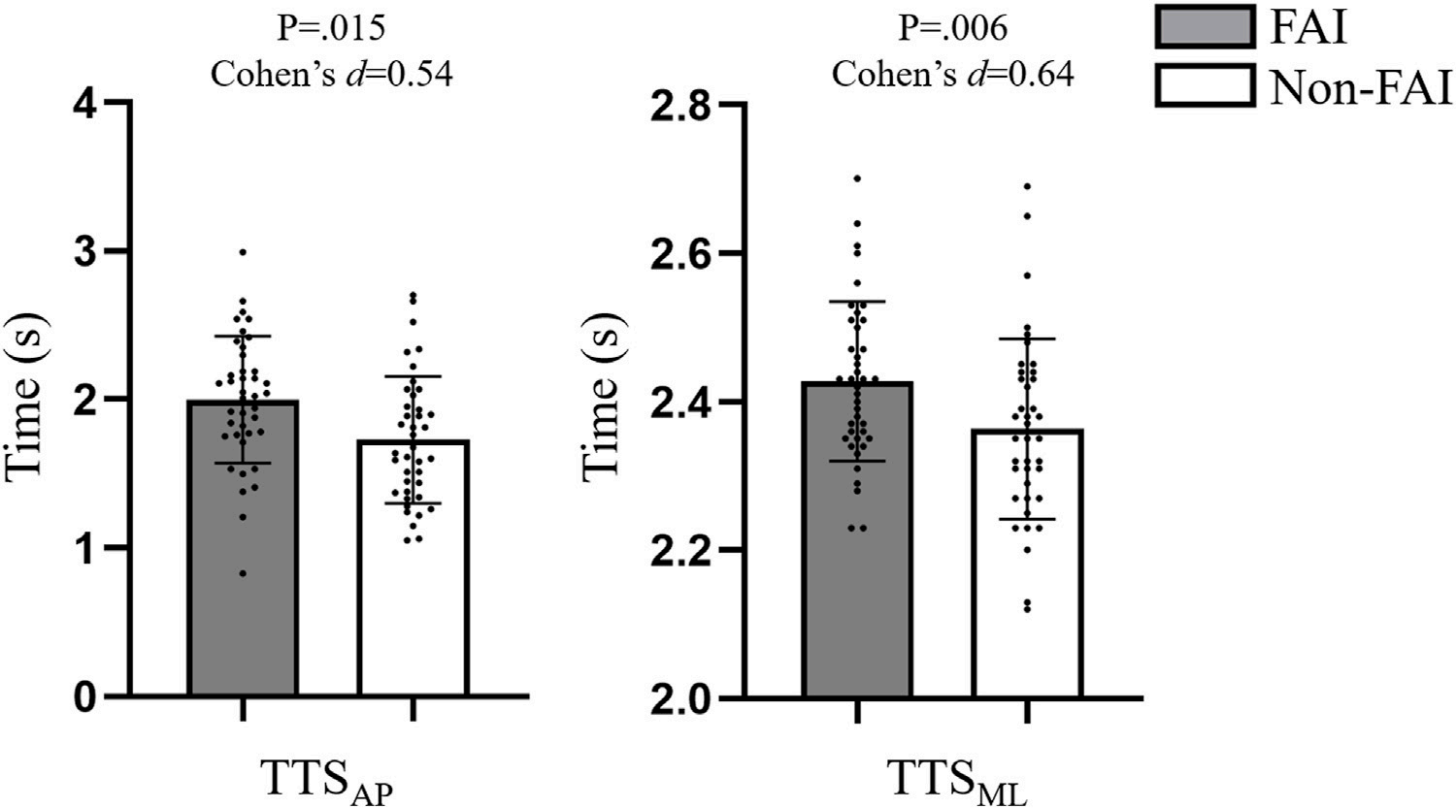
Comparisons of TTS among individuals with and without FAI. TTS: time to stabilization; AP: anterior-posterior direction; ML: medial-lateral direction.

Shapiro-Wilk test results showed the nonnormal distribution of all proprioception variables and strength during ankle dorsiflexion. The descriptive characteristics of the TTS are shown in Figure 2. Individuals with FAI had longer TTS_AP_ and TTS_ML_ than those without FAI.

The descriptive characteristics of proprioception and strength are shown in Table 2. Compared with individuals without FAI, those with FAI had larger proprioception thresholds and less strength during ankle plantarflexion/dorsiflexion and inversion/eversion.

**TABLE 2:** Descriptive characteristics of postural stability, proprioception, and strength.

	FAI (n = 40)	Non-FAI (n = 40)	p	Cohen’s *d*	ɳ^2^
**Proprioception (˚)**					
Ankle plantarflexion	1.03 [0.71,1.50]	0.73 [0.58, 1.15]	**.001**	-	0.139
Ankle dorsiflexion	1.12 [0.75, 1.72]	0.70 [0.52, 1.14]	**<.001**	-	0.214
Ankle inversion	2.45 [1.61, 3.65]	1.45 [1.16, 1.90]	**<.001**	-	0.194
Ankle eversion	2.59 [1.53, 3.85]	1.50 [1.03, 1.99]	**<.001**	-	0.204
Strength (N*m/kg)					
Ankle plantarflexion^a^	0.90 ± 0.33	1.09 ± 0.34	**.012**	0.56	-
Ankle dorsiflexion	0.22 ± 0.08	0.28 [0.23, 0.38]	**.002**	-	0.125
Ankle inversion^a^	0.21 ± 0.09	0.25 ± 0.07	**.017**	0.49	-
Ankle eversion^a^	0.11 ± 0.04	0.14 ± 0.03	**.001**	0.84	-

Data are presented as mean ± SD (normal distribution) or median [interquartile range] (nonnormal distribution). Bold: *p* < .05. The thresholds for effect size (Cohen’s *d*) were as follows: <0.20, trivial; 0.21–0.50, small; 0.51–0.80, medium; >0.81, large. The thresholds for effect size (ɳ^2^) were as follows: 0.01–0.059, small; 0.06–0.14, medium; >0.14, large.

^a^ Analyzed by Independent t-tests, others by Mann-Whitney U tests.

The correlations of proprioception and strength to TTS are shown in Table 3. Among the individuals with FAI, the strength during ankle plantarflexion was moderately correlated with TTS_AP_. Among the individuals without FAI, the strength during ankle plantarflexion/dorsiflexion was moderately correlated with TTS_AP_, the proprioception during ankle inversion/eversion was moderately correlated with TTS_ML_, and the strengths during ankle eversion were moderately correlated with TTS_ML_.

**TABLE 3:** Correlations of proprioception and strength with TTS.

		Variables	FAI				Non-FAI			
			TTS_AP_		TTS_ML_		TTS_AP_		TTS_ML_	
			r	p	r	p	r	p	r	p
Ankle proprioception (˚)	F1	Ankle plantarflexion	−0.136	0.403	−0.188	0.247	−0.012	0.943	−0.124	0.445
		Ankle dorsiflexion	−0.092	0.572	−0.235	0.144	−0.029	0.858	0.060	0.175
	F2	Ankle inversion	−0.009	0.956	−0.088	0.289	0.078	0.635	0.327	0.040
		Ankle eversion	−0.106	0.513	−0.094	0.562	0.102	0.531	0.354	0.025
Ankle strength (N*m/kg)	F3	Ankle plantarflexion	−0.409^a^	0.009	0.183^a^	0.258	−0.348^a^	0.028	−0.268^a^	0.094
		Ankle dorsiflexion	−0.238^a^	0.140	0.116^a^	0.477	−0.473	0.002	−0.050	0.758
	F4	Ankle inversion	−0.173^a^	0.286	0.135^a^	0.407	−0.189^a^	0.242	−0.195^a^	0.229
		Ankle eversion	−0.125^a^	0.441	0.157^a^	0.334	−0.125^a^	0.443	−0.479^a^	0.002

TTS: time to stabilization; AP: anterior-posterior direction; ML: medial-lateral direction. Shaded cells represent moderate correlation coefficients (.3–.5).

^a^ Analyzed by Pearson correlation, others by Spearman correlation. F: factor. F1, proprioception during ankle plantarflexion/dorsiflexion. F2, proprioception during ankle inversion/eversion. F3, strength during ankle plantarflexion/dorsiflexion. F4, strength during ankle inversion/eversion.

The factor loadings for all the variables of proprioception and strength are shown in Table 4. Factor 1 (F1), factor 2 (F2), factor 3 (F3), and factor 4 (F4) were the summaries of proprioception during ankle plantarflexion/dorsiflexion and inversion/eversion and strength during ankle plantarflexion/dorsiflexion and inversion/eversion, with a Kaiser Meyer Olkin value of 0.760 and sphericity of <0.001.

**TABLE 4:** Factor loadings of the variables among the categories of proprioception and strength.

		FAI group				Non-FAI group			
		F1	F2	F3	F4	F1	F2	F3	F4
Ankle proprioception	Plantarflexion	0.971	--	--	--	0.900	--	--	--
	Dorsiflexion	0.980	--	--	--	0.910	--	--	--
	Inversion	--	0.952	--	--	--	0.937	--	--
	Eversion	--	0.959	--	--	--	0.911	--	--
Ankle strength	Plantarflexion	--	--	0.864	--	--	--	0.728	--
	Dorsiflexion	--	--	0.805	--	--	--	0.849	--
	Inversion	--	--	--	0.876	--	--	--	0.862
	Eversion	--	--	--	0.880	--	--	--	0.810

F: factor, --: factor loading <0.500.

The equation for multivariable regression among individuals with FAI is:

$${\text{TTS}}_{\text{AP}}=2.42\;-\;\left(0.042\times F3\right)$$ (4)

In Eq. 4, adjusted *r* ^2^ = 0.414, p_F3_ = 0.014, and β_F3_ = 0.388.

The equations for multivariable regression among individuals without FAI are:

$${\text{TTS}}_{\text{AP}}=2.36\;-\;\left(0.051\times F3\right)$$ (5)

$${\text{TTS}}_{\text{ML}}=1.72+\left(0.134\times F2\right)\;-\;\left(0.138\times F4\right)$$ (6)

In Eq. 5, adjusted *r* ^2^ = 0.432, p_F3_ = 0.008, and β_F3_ = 0.420.

In Eq. 6, adjusted *r* ^2^ = 0.458, p_F2_ = 0.040, p_F4_ = 0.035, β_F2_ = 0.313, and β_F4_ = 0.322.

## 4 Discussion

This study compared postural stability, proprioception, and strength between individuals with and without FAI and investigated the correlations of proprioception and strength with AP and ML postural stability. The results partially approved hypothesis # 1 and rejected hypothesis # 2. The individuals with FAI exhibited worse postural stability and proprioception and less strength than those without FAI. Both groups demonstrated the correlations of strength with AP postural stability. The individuals without FAI demonstrated the correlations of proprioception and strength with ML postural stability, but no such correlations were detected among those with FAI.

The results demonstrated that the individuals with FAI exhibited worse postural stability and proprioception and less strength than those without FAI. The impaired postural stability among individuals with FAI aligns with previous studies (Ross et al., 2009), the reason may line in the proprioception and neuromuscular control deficits after recurrent sprains (Hertel and Corbett, 2019). Several studies (Sousa et al., 2017; Xue et al., 2021) consistently reported that individuals with FAI demonstrated worse proprioception than those without FAI. This condition may be caused by the damaged proprioceptors, such as muscle spindles, Pacinian corpuscles, and Ruffini corpuscles, due to recurrent ankle sprains (Xue et al., 2021). The finding that individuals with FAI have less strength was consistent with a previous study (Arnold et al., 2009), which stated that this condition is a result of damage or atrophy of the muscles around the ankle (Khalaj et al., 2020). Decreased proprioception may also be a potential cause of decreased muscle strength. When proprioception has deteriorated, the muscles are unable to receive accurate feedback, leading to a decrease in the precision of muscle control around the ankle, which in turn affects muscle strength (Hertel and Corbett, 2019).

Our results showed the correlations between strength and AP postural stability among individuals with and without FAI. The ankle dorsiflexors and plantarflexors could control the backward and forward movements of the body to prevent the center of mass from moving out of the posterior and anterior edges of the support base (Svoboda et al., 2019), thus helping maintain and restore AP postural stability during movements. In addition, the contraction of dorsiflexors and plantarflexors contributes to the dynamic stability at the ankle and the postural stability of the body and reduces the impact of GRF on the body (Park et al., 2019; Khalaj et al., 2021). Although their strength decreased due to recurrent ankle sprains, both groups still demonstrated the correlations between strength and AP postural stability, suggesting that strength continues to play a crucial role in AP postural stability among individuals with FAI.

One interesting finding of this study is that proprioception and strength contributed to ML postural stability among individuals without FAI but not among those with FAI. The CNS utilizes proprioceptive feedback to estimate the position of the lower limbs and then activates the appropriate muscles to facilitate rapid lateral adjustments to maintain ML postural stability (Rankin et al., 2014). Compared with those without FAI, the weaker correlation among individuals with FAI infers that they may rely less on their deteriorated proprioception and decreased strength to maintain ML postural stability. Recurrent ankle sprains could disrupt proprioception receptors and lead to muscle atrophy, which affects the perception of ankle proprioception and the development of joint force and leads to impaired neuromuscular control during joint movement (Hertel and Corbett, 2019; Hopkins et al., 2019). It can be inferred that once proprioception and strength deteriorated to a certain point, they could not provide meaningful functional assistance to the ML postural stability. Given that most ankle sprains occur in the ML direction (mostly during ankle inversion) (Herzog et al., 2019), the worse proprioception and less strength might reasonably explain the increased risk of ankle sprains among individuals with FAI. On the basis of the above inference and the confirmation of the proprioception and strength decline among individuals with FAI, it is reasonable to assume that the rehabilitation of proprioception and strength could be keys to prevent recurrent ankle sprains among individuals with FAI.

This study has several limitations. First, some potential factors, such as plantar tactile sensation, were not measured. Nevertheless, tactile sensation was transmitted by small diameter type III sensory neurons, which are slower and weaker than other sensory neurons (Li et al., 2019), so the effect of tactile sensation is limited during dynamic tasks. Second, as a cross-sectional study, the causal relationships between proprioception and strength to postural stability cannot be explained. Longitudinal studies could help deepen our understanding of how postural stability is affected by proprioception and strength.

## 5 Conclusion

Individuals with FAI have worse postural stability and proprioception and less strength than those without FAI. Their proprioception and strength decreased to a point where they could not provide sufficient functional assistance to the ML postural stability. Improvements in proprioception and strength may be keys to prevent recurrent ankle sprains among individuals with FAI.

## Data Availability

The original contributions presented in the study are included in the article/Supplementary material, further inquiries can be directed to the corresponding authors.
